# An external pilot cluster randomised controlled trial of a theory-based intervention to improve appropriate polypharmacy in older people in primary care (PolyPrime): study protocol

**DOI:** 10.1186/s40814-021-00822-2

**Published:** 2021-03-19

**Authors:** Audrey Rankin, Cathal A. Cadogan, Heather E. Barry, Evie Gardner, Ashley Agus, Gerard J. Molloy, Ashleigh Gorman, Cristín Ryan, Claire Leathem, Marina Maxwell, Gerard J. Gormley, Alan Ferrett, Pat McCarthy, Tom Fahey, Carmel M. Hughes, On behalf of the PolyPrime team

**Affiliations:** 1 School of Pharmacy, Queen’s University Belfast, 97 Lisburn Road, Belfast, BT9 7BL UK; 2 School of Pharmacy and Biomolecular Sciences, Royal College of Surgeons in Ireland, Dublin, Ireland; 3 Northern Ireland Clinical Trials Unit, Belfast, UK; 4 School of Psychology, National University of Ireland, Galway, Ireland; 5 School of Pharmacy and Pharmaceutical Sciences, Trinity College Dublin, Dublin, Ireland; 6 Northern Ireland Clinical Research Network (Primary Care), Belfast, UK; 7 School of Medicine, Dentistry and Biomedical Sciences, Queen’s University Belfast, Belfast, UK; 8 Public Involvement Enhancing Research, Belfast, Northern Ireland UK; 9 Donegal Volunteer Centre, Donegal, Ireland; 10 Department of General Practice, Royal College of Surgeons in Ireland, Dublin, Ireland

**Keywords:** Polypharmacy, Behaviour change, Primary care, General practice, Complex intervention, Pilot study, Process evaluation, Older people, Prescribing

## Abstract

**Background:**

The use of multiple medications (polypharmacy) is a concern in older people (≥65 years) and is associated with negative health outcomes. For older populations with multimorbidity, polypharmacy is the reality and the key challenge is ensuring appropriate polypharmacy (as opposed to inappropriate polypharmacy). This external pilot cluster randomised controlled trial (cRCT) aims to further test a theory-based intervention to improve appropriate polypharmacy in older people in primary care in two jurisdictions, Northern Ireland (NI) and the Republic of Ireland (ROI).

**Methods:**

Twelve GP practices across NI (*n*=6) and the six counties in the ROI that border NI will be randomised to either the intervention or usual care group. Members of the research team have developed an intervention to improve appropriate polypharmacy in older people in primary care using the Theoretical Domains Framework of behaviour change. The intervention consists of two components: (1) an online video which demonstrates how a GP may prescribe appropriate polypharmacy during a consultation with an older patient and (2) a patient recall process, whereby patients are invited to scheduled medication review consultations with GPs. Ten older patients receiving polypharmacy (≥4 medications) will be recruited per GP practice (*n*=120). GP practices allocated to the intervention arm will be asked to watch the online video and schedule medication reviews with patients on two occasions; an initial and a 6-month follow-up appointment. GP practices allocated to the control arm will continue to provide usual care to patients. The study will assess the feasibility of recruitment, retention and study procedures including collecting data on medication appropriateness (from GP records), quality of life and health service use (i.e. hospitalisations). An embedded process evaluation will assess intervention fidelity (i.e. was the intervention delivered as intended), acceptability of the intervention and potential mechanisms of action.

**Discussion:**

This pilot cRCT will provide evidence of the feasibility of a range of study parameters such as recruitment and retention, data collection procedures and the acceptability of the intervention. Pre-specified progression criteria will also be used to determine whether or not to proceed to a definitive cRCT.

**Trial registration:**

ISRCTN, ISRCTN41009897. Registered 19 November 2019. ClinicalTrials.gov, NCT04181879. Registered 02 December 2019.

**Supplementary Information:**

The online version contains supplementary material available at 10.1186/s40814-021-00822-2.

## Background

In the UK (UK) and Ireland, the number of older people (aged ≥65 years) is growing and estimated to reach nearly a quarter of the population by 2035 [1]. The use of multiple medications (polypharmacy) in this population has been described as the ‘single most important health care intervention in the industrialised world’ [2]. Historically, polypharmacy was viewed negatively because of potential medication safety-related risks; however, there is growing recognition that polypharmacy may be entirely appropriate, particularly with the increasing prevalence of multimorbidity (≥2 long-term conditions) and guidelines advocating the use of more than one medication in long-term condition management, e.g. hypertension [3]. Polypharmacy is increasingly seen as ‘potentially problematic rather than always inappropriate’. Thus, assessments of prescribing appropriateness should extend beyond the number of medications prescribed and consider co-morbidities, in differentiating between ‘many’ medications (appropriate polypharmacy) and ‘too many’ medications (inappropriate polypharmacy) [4]. The concept of ‘appropriate polypharmacy’ recognises that patients can benefit from multiple medications if prescribing is evidence-based, reflects patients’ clinical conditions and preferences, and considers potential drug interactions [5].

Based on the Medical Research Council (MRC) guidance on complex interventions [6, 7], a theory-based intervention has been developed to improve appropriate polypharmacy in older people in primary care [8, 9]. The intervention was developed in Northern Ireland (NI) following Theoretical Domains Framework (TDF)-based semi-structured interviews with General Practitioners (GPs) and community pharmacists [10, 11]. Interviews were analysed using the TDF to identify theoretical domains perceived as barriers and facilitators to prescribing and dispensing appropriate polypharmacy. These domains were then mapped to four behaviour change techniques (BCTs) from an established taxonomy [12, 13] and embedded in the intervention as the active components [10]. The intervention package consisted of a short online video (lasting approximately 11.5 min) which demonstrated how a GP may prescribe appropriate polypharmacy during a typical consultation with an older patient (BCT: ‘Modelling or demonstrating of behaviour’) [14]. The video also included feedback from both a practising GP and a simulated patient emphasising the positive outcomes of the consultation (BCT: ‘Salience of consequences’). A patient recall process was included as a complementary intervention component, whereby patients were invited to scheduled medication review consultations with GPs. In addition, explicit plans were made at practice staff meetings of when and how GPs would ensure that target patients were prescribed appropriate polypharmacy (BCT: ‘action planning’) [14]. Reception staff then scheduled consultations for eligible patients and prompted GPs to review patients’ medications when they presented at the practice by notifying GPs that the patients were attending a scheduled consultation (BCT: ‘prompts/cues’) [14].

Preliminary testing of the intervention in a small-scale feasibility study was completed in two general practices in NI (ISRCTN18176245) [15]. The intervention was considered usable and acceptable by GPs. Patient feedback on the scheduled consultations was positive, and patients welcomed the opportunity to have their medications reviewed [15]. However, the lack of detail in data extracted from patient medical records meant that an assessment of medication appropriateness using the Screening Tool of Older People’s potentially inappropriate Prescriptions (STOPP)/Screening Tool to Alert doctors to Right Treatment (START) criteria [16] was not possible.

Subsequent research has led to a three-phase project which will incorporate an intervention refinement phase (phase 1), an external pilot randomised controlled trial with an embedded health economic analysis (phase 2; described within this protocol) and a mixed methods process evaluation (phase 3; see the ‘Process Evaluation’ section). The current study (phase 2) will address elements of uncertainty arising from the previous feasibility study and progress the development of the intervention further, through testing in a larger external pilot study in GP practices within two different health care systems in NI and the Republic of Ireland (ROI), including the six counties of Northern Ireland and the border region of the ROI (counties of Cavan, Donegal, Leitrim, Louth, Monaghan and Sligo). There is evidence to suggest that prescribing patterns in NI and the ROI are similar [17, 18], and overall, general practice does not differ markedly between the two jurisdictions [19]. However, there may be other more subtle differences (e.g. context) that are important to intervention implementation [20]. Understanding these differences will be the key to determining whether the intervention package (which was designed in NI) can be implemented in the two jurisdictions or if modifications are required.

Phase 1 of this project, an intervention refinement exercise, was completed by interviewing GPs from 12 GP practices in the six border counties of the ROI outlined above. During the interviews, GPs were asked about their views of polypharmacy in older people and their approach to prescribing for this age group. The intervention package was described in more detail and GPs were shown the existing video component. GPs were then asked to comment on the content of the intervention package, mode of delivery, relevance to practice, and to suggest any changes they felt would be required. Based on the findings, additional educational slides have been incorporated into the video that highlight key issues which GPs should consider when conducting the medication reviews and where they can go for further information (see the ‘Intervention specification’ section) [21].

This protocol describes the rationale, methods and analysis plan for phase 2, consisting of an external pilot cluster randomised controlled trial (cRCT) which will test the refined intervention, with an embedded process evaluation and health economic analysis.

## Methods/design

### Aim

The primary aim of this study is to assess the feasibility of a definitive cRCT of the effectiveness and cost-effectiveness of the PolyPrime intervention in primary care in NI and the ROI. The objectives of the study are as follows:

1. Test approaches to sampling, recruitment and retention of GP practices and patients
2. Test the feasibility of using medication appropriateness as the primary outcome in a future cRCT
3. Identify the resources used in the set-up and delivery of the intervention and their associated costs
4. Assess the feasibility of a future cost-effectiveness analysis
5. Further validate the Medication-Related Burden Quality of Life (MRB-QoL) tool
6. Obtain estimates of effect size between groups, cluster size and intraclass correlation coefficients to inform the sample size calculation for a full cRCT
7. Identify the intervention’s likely mechanism of action
8. Assess if the intervention is delivered and received as intended (intervention fidelity)

### Study design

This study is an external pilot cRCT, applying the definition developed by Eldridge and colleagues, i.e. randomised, conducted in advance of a future definitive RCT, and primarily aimed to assess feasibility [22]. Ethical approval was granted by the North of Scotland (REC reference: 19/NS/0100) and the Irish College of General Practitioners (ICGP) Research Ethics Committees (RECs). This study protocol has been reported according to the SPIRIT (Standard Protocol Items: Recommendations for Interventional Trials) 2013 statement [23] (see Additional file 1: SPIRIT figure; and Additional file 2 for a completed SPIRIT checklist).

### Setting

The study will be conducted in 12 GP practices in NI and the border counties of the ROI (Donegal, Leitrim, Sligo, Cavan, Monaghan and Louth), with the aim of recruiting one GP practice per county.

In NI, primary care services (e.g. GP visits) are publicly funded and provided free at the point of delivery through the National Health Service (NHS), while in the ROI, primary care services are publicly and privately funded. Those aged ≥70 years old are entitled to a General Medical Services (GMS) card, which provides free access to primary care services, albeit with a small co-payment in place on prescription medications.

### GP practice recruitment

A randomised list of GP practices in NI and the ROI border counties has been compiled using publicly available information from the Health and Social Care Business Services Organisation and the Irish Medical Directory, respectively. In the first instance, up to 15 GP practices per county will be contacted via a letter seeking expressions of interest. Those who return the reply slip will be contacted by one of the researchers to provide additional information about the study. The GP practices will be given sufficient time to decide whether they wish to participate (within 10 days), after which the researchers will telephone the practices to try to recruit them.

If the required number of GP practices has not been achieved, a second stage of GP practice recruitment will be facilitated by research nurses from the Northern Ireland Clinical Research Network (NICRN – Primary Care) and Trinity College Dublin (TCD). Research nurses will telephone the practice manager or lead GP in each practice to determine their interest in receiving information about the study. With their agreement, study information will either be posted or emailed to the practice and they will be given sufficient time to decide whether they wish to participate (within 10 days). If selected practices decline to participate, the next practice on the randomised list will be contacted. In phase 1 of this project, recruitment of GP practices across the six ROI border counties was challenging due to the limited number of GP practices within some of the counties. Therefore, if the original target of recruiting one GP practice from each of the counties is not met, more than one practice per country may be recruited, with the aim of providing a final sample of six practices per jurisdiction, i.e. NI and ROI.

GP practices will be eligible to participate if they provide written informed consent and research governance sign-off, have a stable internet service in order to access the video and are not currently participating in other studies related to medicines management in older people. Individual GP participants will receive a certificate of participation which can be used as part of their continuing professional development. GP practices will also be given an honorarium of £855/€1000 as compensation for the time and resources associated with study participation. An additional £92/€108 (intervention arm) or £46/€54 (control arm) will be paid to GP practices for each patient successfully recruited into the study. These rates were based on what had been used in previous studies and the availability of funding within the grant supporting the study. Intervention arm practices will be paid more than the control arm practices per patient to reflect the time commitment required on behalf of GPs and practice staff.

### Patient screening and recruitment

Once 12 GP practices have been recruited, each will be asked to screen patient records to identify and filter potentially eligible patients with the goal of recruiting approximately 10 patients per practice. As this is a pilot study, a formal sample size calculation is not required; however, based on previous research conducted by members of the research team [11], 10 patients per site (120 patients in total) have been deemed sufficient to meet the aims and objectives of the study.

With the support of the aforementioned research nurses, GP practice staff will randomly screen patients by either using available technology to generate random numbers or by hand screening the random patients on the list. The following patient inclusion criteria will apply: aged 70 years or older, receiving four or more regular medications (i.e. prescribed for more than 3 months), resident in the community, in receipt of a valid GMS card in the ROI, or registered for NHS primary care services in NI and have been attending the practice for a minimum of 12 months. Patients will be excluded if they are care home residents, cognitively impaired (as determined by the GP and/or practice staff), have a terminal illness or are currently involved in other Investigational Medicinal Product or medicines management studies.

Invitation letters will be mailed to eligible patients from each practice along with an information sheet, consent form and baseline questionnaires (see the ‘Outcome data collection’ section). The letter will direct interested patients to return completed consent forms and questionnaires or contact the research team if they would like to take part in the study. Invitation letters will be posted in batches of 25 until the required number of patients is recruited. Each GP practice will also display a patient recruitment poster in their practice waiting areas to promote the study and aid recruitment.

Patient screening and recruitment began on January 22, 2020, at the time of submission of this paper, patient screening and recruitment had commenced across nine sites.

### Randomisation

Eligible GP practices will be allocated to intervention or control group by a statistician using an automated randomisation system. Practices will be randomised on a 1:1 allocation ratio stratified by country (i.e. NI or ROI). The randomisation sequence will be concealed using an automated randomisation system and will only be accessible to the statistician.

### Intervention specification

As discussed above, the existing intervention package consists of two main components; an online video which demonstrates how GPs can improve appropriate polypharmacy during typical consultations with older patients, and a patient recall process, whereby patients are invited to medication review consultations with GPs [4, 15]. The video component seeks to enable GPs to use available time more efficiently by demonstrating how appropriate polypharmacy can be prescribed during routine consultations with older patients (BCT: Modelling or demonstrating of behaviour), rather than introducing new behaviours or tasks for GPs to perform. In addition, the video emphasises the potentially positive consequences of performing this behaviour (BCT: Salience of consequences) (see Table 1). The intervention seeks to introduce small, but potentially sustainable changes in GPs’ current clinical practice aimed at improving prescribing for older people. Due to expected clinical heterogeneity amongst the target patient population, it will not be possible to standardise the medication review consultation.

**Table 1:** Behaviour change techniques (BCTs) operationalised as part of the PolyPrime intervention

Behaviour change technique (BCT)	BCT delivery as part of the PolyPrime intervention
Action planning	During GP practice staff meetings, GPs will plan to perform medication reviews on a specified date when patients meeting inclusion criteria present at the practice for a scheduled appointment.
Prompts/cues	GPs will be prompted by the receptionist/practice manager to perform medication reviews with older patients meeting inclusion criteria when patients present for a scheduled appointment.
Modelling or demonstrating of behaviour	GPs will be provided with a video demonstration of how to perform a medication review with an older patient who is receiving polypharmacy.
Salience of consequences	As part of the video demonstration of how to perform a medication review, feedback will be included from the GP and ‘patient’ to emphasise the potentially positive consequences of performing the review.

Based on the findings from phase 1, additional educational slides have been incorporated into the video that highlight key issues which GPs should consider when conducting the medication reviews, such as the most common instances of inappropriate prescribing, the tools used to support medication reviews [i.e. STOPP/START 16] and NO TEARS (Need and indication; Open questions; Tests and monitoring; Evidence and guidelines; Adverse events; Risk reduction or prevention; Simplification and switches) tool [24]) and where to go for further information [i.e. National Institute for Health and Care Excellence (NICE) guidance for medicines optimisation [21, 25].

To facilitate the patient recall process, two complementary intervention components were also included which involve GPs making explicit plans at weekly practice staff meetings of when and how they would ensure that target patients were prescribed appropriate polypharmacy (BCT: Action planning) and GPs receiving prompts from reception staff to carry out this plan when target patients arrive at the practice (BCT: Prompts/cues).

### Intervention arm

Once practices have been allocated to the intervention arm, the researchers will visit each practice to train GPs and practice staff on how to implement the intervention package. Recruited GPs will be asked to watch the online video by logging into a dedicated project website, which they will be able to access throughout the intervention delivery phase using individual usernames and passwords. As outlined above as part of the patient recall process, each GP practice will be asked to schedule medication review appointments (on two occasions; an initial medication review and a 6-month follow-up appointment) with the 10 recruited patients. GPs will discuss scheduling medication review appointments during a weekly practice staff meeting and will be prompted by reception staff to perform these when patients arrive at the practice (see Table 1). Based on the feedback received in phase 1, practice staff will also receive an information sheet outlining their involvement in relation to scheduling medication review appointments and prompting the GPs upon the patient’s arrival.

### Usual care (control) arm

GP practices allocated to the control arm will continue to provide usual care for patients. The GPs will not be provided with access to the online video and will not be required to conduct medication reviews. Usual care, defined as prescribing as per their standard practice, may vary depending on the GP practice. Given the differences in primary care provision, GP practices will be asked to provide a brief overview of their current prescribing practice (usual care) for older patients receiving polypharmacy, if medication reviews are conducted and by whom (e.g. in NI, medication reviews may be routinely undertaken by a practice-based pharmacist as part of standard primary care services). Randomisation to control may also affect retention which is important in informing decisions about progression to a future larger study. The usual care group will be given access to the intervention resources at a later date once the study is complete.

### Outcomes

Outcomes to be collected in this study relate to feasibility parameters associated with recruitment and retention of patients and practices, and data collection. We will also explore the assessment of appropriateness of prescribing, health-related quality of life and a health economic analysis. Approaches to data collection are outlined below.

### Outcome data collection

We had originally planned to collect outcome data at baseline, 6 months (before the second review for intervention patients) and 12 months post-baseline for patients in both arms of the study. However, due to the ongoing impact of the COVID-19 pandemic, we have had to revise the follow-up time points. Outcome data will be collected at baseline, 6 months (before the second review for intervention patients) and 9 months post-baseline from the intervention arm patients. The follow-up time points for the control arm will be based on the average length of time from the completion of baseline data collection to 6 and 9 months post-initial medication review in the intervention arm. The pilot study will assess the feasibility of study procedures including recruitment and retention of both practices and patients and data collection (patient self-report and GP records). In terms of more definitive outcomes, members of the research team have developed a core outcome set for trials aimed at improving appropriate polypharmacy in older people in primary care [26]. The seven highest-ranked outcomes were serious adverse drug reactions, medication appropriateness, falls, medication regimen complexity, quality of life, mortality and medication side effects. One of the issues with many core outcome sets is that multiple outcomes are identified, and further refinement is needed to select a manageable number of outcomes to include in a study. For the purpose of this external pilot cRCT, the research team have agreed to focus on medication appropriateness and quality of life.

In order to assess medication appropriateness, patient data [including medical history, clinical conditions, biochemical data (i.e. test results) and prescribed medications] will be collected on a study-specific case report form (CRF) from GP records. An assessment of prescribing appropriateness will then be made by pharmacists on the research team using the STOPP/START criteria [16]. Health-related quality of life (HRQoL) will be measured using the EQ-5D-5L questionnaire [27] and the medicine-related burden quality of life (MRB-QoL) tool [28]. In addition, a validation exercise will be undertaken to check the psychometric properties of the MRB-QoL tool. Firstly, a confirmatory factor analysis will be performed to evaluate construct validity of the instrument using baseline data. Secondly, the sensitivity and responsiveness of the MRB-QoL tool will be evaluated using data collected at the time points indicated above.

For the health economic analysis and to assess the feasibility of embedding a cost-effectiveness analysis in a future definitive trial, patients’ use of health services (e.g. primary, secondary, social care) will be recorded in two different ways (patient self-report and GP records) and then compared to assess the most appropriate method. A diary will be given to patients to allow them to record their health service use prospectively. Baseline questionnaires (measuring quality of life and health service use) will be sent to patients along with the initial invitation letters. Postal questionnaires will be sent at 6 months (before intervention arm patients’ second medication review), 9 months post-initial medication review in the intervention arm and at the equivalent time points for the control group as outlined above. Patients will be telephoned as a reminder and given the opportunity to complete questionnaires via telephone if preferred.

### Blinding

Due to the nature of the intervention package (i.e. access to the video and patient recall), we will be unable to blind the GPs, practice staff or patients. Research nurses collecting patient data (including medical history, clinical conditions, biochemical data (i.e. test results) and prescribed medications) from GP records will be blinded at baseline. However, due to the nature of the intervention, we will be unable to blind research nurses for data collection. Members of the research team, who are pharmacists, will undertake an assessment of medication appropriateness using the data collected from GP records and will be blinded to the allocation of the intervention and control arms.

### Process evaluation

**Fig. 1:**
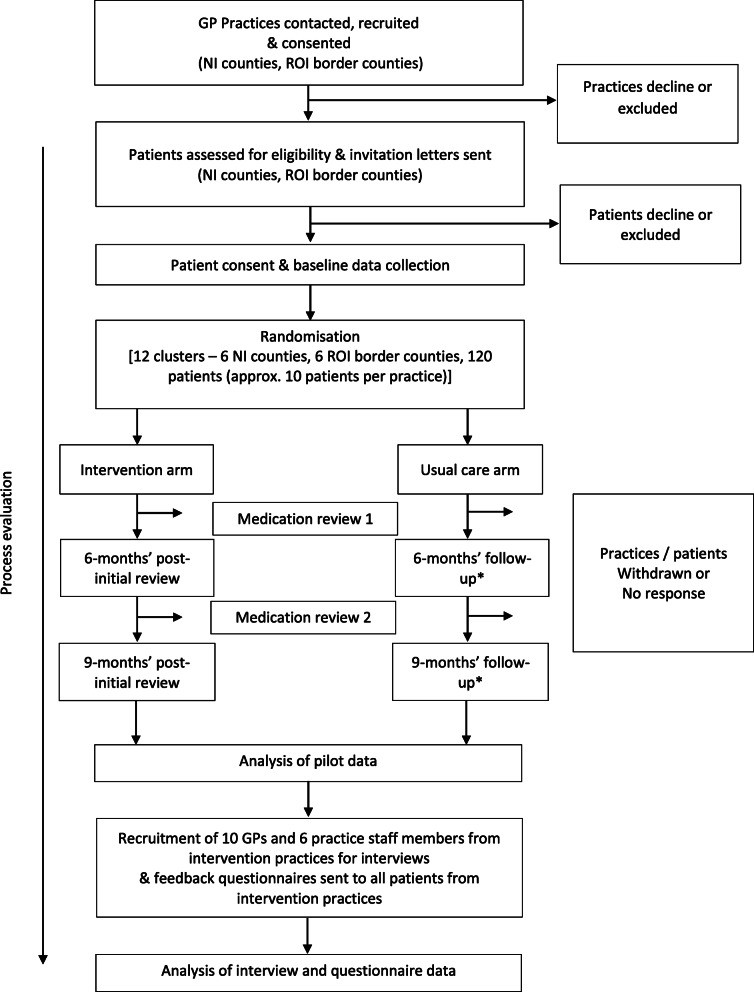
Overview of the PolyPrime study. *The follow-up time points for the control arm will be based on the average length of time from the completion of baseline data collection to 6 and 9 months post initial medication review in the intervention arm

A mixed methods process evaluation (phase 3) will be embedded to run alongside and following completion of the intervention (see Fig. 1) to assess (1) intervention fidelity (i.e. was the intervention delivered, received and enacted as intended); (2) acceptability of the intervention; and (3) potential mechanisms of action (to explore how the intervention might impact upon GPs’ prescribing behaviour). Further details of the protocol for the process evaluation will be published in a separate paper.

### Progression criteria

This external pilot study will also be used to determine progression to a definitive cRCT of the PolyPrime trial or if further modifications are warranted. The progression criteria outlined in Table 2 will consider recruitment and retention of GPs and patients, intervention fidelity (i.e. was the intervention delivered as intended) and completeness of outcome data. These criteria have been developed in conjunction with the Trial Steering Committee (TSC) and Project Management Group, following a traffic light assessment system (red = stop; amber = amend; green = go) established by Avery et al. [29]. The cut-off points for stop/amend/go criteria have been based on work published by Borelli et al. [30], whereby when ≥80% of the target is met, the criteria meet the ‘go’ thresholds, when 50% of the target is met the criteria meet the ‘amend’ thresholds or when <50% of the target is met, the criteria meet the ‘stop’ thresholds. Once data are available for all the criteria listed in Table 2, the final decision regarding progression of the PolyPrime pilot study towards a definitive cRCT will be made by the TSC using the guidelines outlined in Table 3.

**Table 2:** Progression criteria for the PolyPrime study

Criteria	Data source(s)	Progression criteria		
		Stop (unless there are clear and modifiable contextual or design issues that account for this^**a**^)	Amend	Go
GP practice recruitment	Recruitment records held by research nurse(s)	If ≤5 GP practices are recruited within 8 months	If 6–9 GP practices are recruited and/or it takes longer than predicted (6-8 months)	If ≥10 GP practices are recruited to take part in ≤ 6 months
GP practice retention	Retention records held by research nurse(s)	If ≤5 GP practices are retained for the required period	If 6-9 GP practices can be retained for the required period	If ≥10 GP practices can be retained for the required period
Patient recruitment	Recruitment records held by Research Fellow/Assistant	If ≤59 patients are recruited within 5 months^b^	If 60–95 patients are recruited within 5 months^b^	If ≥96 patients are recruited within 5 months^b^
Patient retention	Retention records held by Research Fellow/Assistant	If ≤49% of patients are retained for the required period	If 50–79% of patients are retained for the required period	If ≥80% of patients are retained for the required period
Completeness of outcome data	Data collected during the study (case report forms, questionnaires)	≤49% of each patient self-report and GP-reported outcome measure is complete	If 50-79% of each patient self-report and GP-reported outcome measure is complete	If ≥80% of each patient self-report and GP-reported outcome measure is complete

^a^This includes aspects of study and/or data collection procedures that may be modified in advance of a full-scale definitive cRCT

^b^Note that if ethics amendments are made to study recruitment procedures during this time period, the 5-month recruitment period may be extended (up to 8 months’) to enable sufficient time to assess patient recruitment rates

^c^Based on data collected from the 6 intervention arm GP practices

**Table 3:** Progression criteria rules

Stop	Amend	Go
If one or more of the criteria^a^ meet the ‘stop’ thresholds, then the study should not progress towards a definitive cRCT, unless there are clear ‘modifiable’ contextual or design issues (i.e. related to the intervention or study procedures) which have been identified	If one or more of the criteria^a^ meet the ‘amend’ thresholds, then these will be discussed with the TSC to ascertain whether there is enough evidence that sufficient improvements can be made to proceed to a definitive cRCT	If all the criteria^a^ meet the ‘go’ thresholds, then with the appropriate amendments (if needed), the study should proceed towards a definitive cRCT

^a^Based on the criteria listed in Table 2

### Statistical methods

Descriptive statistics will be presented for each group, as appropriate for the scale of measurement of each outcome, i.e. count (percentage) for categorical data, mean (standard deviation) for normally distributed continuous data and median (interquartile range) for skewed or ordinal data. The effect size between intervention and control groups, cluster size and intraclass correlation will be estimated in order to inform the parameters for a sample size calculation for a potential full-scale study.

### Health economic analysis

A cost analysis will be conducted alongside the cRCT to determine the direct and associated costs of conducting the PolyPrime intervention in primary care in NI and the ROI. Costs will be calculated by attaching appropriate unit costs from publicly available sources (e.g. Department of Health National Schedule of Reference Costs). Descriptive statistics will be used to summarise the following: resources used in the set-up and delivery of the intervention and associated costs (e.g. GP and practice staff time input), health service use of patients and associated costs (patient self-report and GP records) and responses in the EQ-5D-5L will be converted to an overall utility score which will be used in a calculation of quality adjusted life years.

A Statistical Analysis and Health Economic Analysis Plan will be written by the study statistician and health economist respectively prior to the final analysis.

### Data management and monitoring

All participants (patients, GPs, practice staff and GP practices) will be given a unique study ID number and data will be anonymised/pseudonymised (e.g. CRFs, questionnaires and interview transcripts). Personal data including consent forms, completed questionnaires or transcripts will be held in a locked filing cabinet, within a locked office on a secure keycode-protected floor of the School of Pharmacy, QUB, the School of Pharmacy and Pharmaceutical Sciences, TCD or the Northern Ireland Clinical Trials Unit (NICTU). Electronic data will be stored within the QUB, TCD or NICTU network space and will be password-protected to ensure confidentiality. Interviews will be digitally recorded (with permission and written, informed consent), transcribed and checked for accuracy. Once transcripts have been checked for accuracy recordings will be deleted and transcriptions stored within QUB or TCD. All participant consent forms, questionnaires, CRFs and transcripts stored at TCD or the NICTU will be transferred to QUB, in line with General Data Protection Regulation (GDPR) guidelines for transferral of data, upon study completion. This will be done via recorded delivery for hard copy data and encrypted email for electronic data. When the study has been completed, the forms and transcripts will be securely stored for 5 years and then destroyed.

The conduct of the trial will be overseen by the TSC. Throughout the trial, the TSC will take responsibility for monitoring and guiding overall progress, scientific standards, operational delivery and protecting the rights and safety of trial participants. The TSC includes an independent Chair, two independent clinicians/trialists and an independent statistician. After consultation with the Sponsor, it was decided that due to the low-risk nature of the trial that a Data Monitoring and Ethics Committee (DMEC) was not needed. A Trial Advisory Group (TAG) will also be established for the overall PolyPrime project, consisting of patients/public, primary care academics, and other key stakeholders in general practice/primary care, e.g. practice managers, prescribing advisors. The TAG will meet face-to-face (or by teleconference) once per year and comment on draft reports, and other forms of communication about the study that will be specifically aimed at key stakeholders such as patient groups. Data will be collected on deaths, hospital admissions and Accident & Emergency (A&E) visits at follow-up timepoints to allow the TSC to monitor the safety of trial patients. The TSC will also monitor serious adverse events (SAEs), which in this study are defined as an inpatient hospitalisation, death, persistent or significant disability or incapacity, life-threatening or is otherwise considered medically significant by the investigator. An SAE reporting form will be completed by a GP (i.e. the GP responsible for overseeing the PolyPrime study within each practice) from both the intervention arm and control arm practices and returned to the researchers on a monthly basis. SAE forms from the intervention arm practices will be clinically assessed by two academic GPs on the research team, after which any SAEs linked to the PolyPrime intervention (i.e. a suspected unexpected serious adverse reaction; SUSARs) in the intervention practices will be reported to the TSC and Sponsor for follow-up.

The investigators will conduct the study in compliance with the protocol given approval/favourable opinion by the relevant RECs. Changes to the protocol may require REC approval prior to implementation, except when modification is needed to eliminate an immediate hazard(s) to patients. The CI and trial team, in collaboration with the sponsor, will submit all protocol modifications to the RECs for review in accordance with the governing regulations. Protocol compliance will be monitored by the trial team who will undertake site visits to ensure that the trial protocol is adhered to and necessary paperwork (e.g. CRFs, patient consent) is being completed appropriately. The findings of this external pilot cRCT will be communicated to all participants, published in relevant journals and presented at conferences.

## Discussion

This study aims to test the feasibility of implementing a theory-based intervention to improve appropriate polypharmacy in older people in primary care in relation to recruitment, retention and study procedures including collecting data on medication appropriateness (from GP records), quality of life and health service use (i.e. hospitalisations). The results will be used to determine whether to progress to a definitive cRCT of the PolyPrime intervention or if further modifications are warranted. A recent Cochrane review highlighted a lack of rigour in the development and evaluation of interventions to improve appropriate polypharmacy in older people in primary care and considerable limitations with current evidence (e.g. risks of bias, insufficient details of intervention implementation) ([31]. It is also now recognised that more time should be spent on intervention development, using a systematic approach that incorporates a sound theoretical basis and involves those who deliver and/or receive these interventions, i.e. HCPs, carers and recipients of care [32].

The current study builds on the existing evidence base by further testing a theory-based intervention, originally developed in NI, in a larger cross-border setting. The external pilot cRCT will primarily aim to assess the feasibility of the PolyPrime intervention [22]. Thus, the proposed external pilot study is not intended to provide definitive results in terms of the effectiveness or cost-effectiveness of the intervention package, but rather aims to assess the feasibility of recruitment and study procedures including collecting data on medication appropriateness (from GP records), quality of life and health service use (i.e. hospitalisations). The effect estimates will provide information for sample size, rather than to assess efficacy and could give some indication of a sample size required for a definitive trial if supported by other published work. The cross-border nature of the study will also enable the investigation of data collection procedures within two differing health care systems and research infrastructures. Furthermore, the use of a cluster randomised design will facilitate the objective comparison of the intervention compared to usual care.

An embedded process evaluation will also explore whether the intervention is delivered as intended, if the various study procedures are acceptable for GPs, practice staff and patients and which components of the intervention are likely to influence GP prescribing behaviour and the potential mechanisms of action that are likely to account for any observed changes. Finally, the economic evaluation will investigate the direct and associated costs of the conducting the PolyPrime intervention in primary care in NI and the ROI. Overall, our findings will inform the feasibility of developing a larger, definitive cRCT of the PolyPrime intervention older people.

### Study status

This study was registered at ISRCTN (10.1186/ISRCTN41009897) on 19 November 2019 and ClinicalTrials.gov (https://clinicaltrials.gov/ct2/show/NCT04181879) on December 2, 2019. At the time of submission of the revised manuscript (NI protocol version 4.0; date, November 20, 2020; ROI protocol version 4.0; date, November 10, 2020), 10 GP practices had been recruited. Patient screening and recruitment had commenced with 56 of 120 patients recruited.

### Study amendments

The coronavirus pandemic has had, and continues to have, a dramatic impact upon primary care services in NI and the ROI. Not only has the pandemic affected on how GP practices provide usual care to patients, it has also affected the way in which medication reviews can be delivered. There has been increased use of technology in primary care with greater volumes of both telephone and video consultations [33]. A key component of the PolyPrime intervention is the patient recall process where GP practices allocated to the intervention arm arrange an initial and a 6-month follow-up medication review consultation with patients. The intervention delivery process has therefore been adapted to mitigate the effects of the pandemic as far as possible. As such, GPs in the intervention arm will be asked to schedule both the initial and 6-month follow-up appointments with consenting patients using a mode of delivery which is appropriate for both the GP and the patient and is in line with the latest government and research governance guidelines relating to social distancing. This would include either telephone or online consultations where a face-to-face consultation is not possible.

In addition, the current pandemic has affected patient recruitment; therefore, the recruitment period has been extended. Data collection points have been changed from 6-month and 12-month post-baseline to 6-month and 9-month post-intervention for intervention patients (i.e. after the patients’ initial medication review). The follow-up time points for the control arm will be based on the average length of time from the completion of baseline data collection to 6 and 9 months post-initial medication review in the intervention arm.

## Supplementary Information

**Additional file 1.** SPIRIT 2013 Checklist.

**Additional file 2.** SPIRIT figure.

## Data Availability

The datasets used and/or analysed during the current study are available from the corresponding author on reasonable request.

## Abbreviations

cRCT: Cluster randomised controlled trial
DMEC: Data Monitoring and Ethics Committee
GMS: General medical services
GP: General practitioner
HRQoL: Health-related quality of life
MRC: Medical Research Council
NHS: National Health Service
NI: Northern Ireland
NICRN: Northern Ireland Clinical Research Network
NICTU: Northern Ireland Clinical Trial Unit
RCT: Randomised controlled trial
REC: Research Ethics Committee
ROI: Republic of Ireland
START: Screening Tool to Alert doctors to Right Treatment
STOPP: Screening Tool of Older People’s potentially inappropriate Prescriptions
TCD: Trinity College Dublin
TDF: Theoretical Domains Framework
TSC: Trial Steering Committee
UK: United Kingdom
