# Supplementary material for: An external pilot cluster randomised controlled trial of a theory-based intervention to improve appropriate polypharmacy in older people in primary care (PolyPrime): study protocol
Source: Pilot Feasibility Stud. 2021 Mar 19;7:77. doi: 10.1186/s40814-021-00822-2 (PMC7977311; doi:10.1186/s40814-021-00822-2)
Supplement: Supplementary file 1 — Additional file 1. SPIRIT 2013 Checklist. [file 40814_2021_822_MOESM1_ESM.doc]

|  | **STUDY PERIOD** | | | | | |
| --- | --- | --- | --- | --- | --- | --- |
|  | **Enrolment** | **Allocation** | **Post-allocation ^a^** | | | **Close-out** |
| **TIMEPOINT** | ***-t_1_*** | **0** | ***t_1_*** | ***t_2_*** | ***t_3_*** | ***t_4_*** |
| **ENROLMENT:** |  |  |  |  |  |  |
| **Eligibility screen** | x |  |  |  |  |  |
| **Informed consent** | x |  |  |  |  |  |
| **Allocation** |  | x |  |  |  |  |
| **INTERVENTIONS:** |  |  |  |  |  |  |
| ***PolyPrime intervention ^b^*** |  |  |  |  |  |  |
| ***Usual care (control)*** |  |  |  |  |  |  |
| **ASSESSMENTS:** |  |  |  |  |  |  |
| ***EQ-5D-5L*** |  |  | x | x | x |  |
| ***MRB-QoL*** |  |  | x | x | x |  |
| ***Health service use (Patient self-report)*** |  |  | x | x | x |  |
| ***Health service use (GP records)*** |  |  | x | x | x |  |
| ***Medication Appropriateness*** |  |  | x | x | x |  |
| ***Patient feedback questionnaire*** |  |  |  |  |  | x |
| ***GP feedback interview*** |  |  |  |  |  | x |
| ***Practice staff feedback interview*** |  |  |  |  |  | x |
| ^a^ t_1_ = baseline; t_2_ = six months (before intervention arm patients’ second medication review); t_3_ = 12 months post-baseline  ^b^ Arrows indicate initial and six-month follow-up medication reviews  **Additional Fig 1**. SPIRIT figure of enrolments, interventions and assessments for the PolyPrime study | | | | | | |
